# Postmenopausal endogenous oestrogens and risk of endometrial cancer: results of a prospective study

**DOI:** 10.1054/bjoc.2001.1704

**Published:** 2001-04

**Authors:** A Zeleniuch-Jacquotte, A Akhmedkhanov, I Kato, K L Koenig, R E Shore, M Y Kim, M Levitz, K R Mittal, U Raju, S Banerjee, P Toniolo

**Affiliations:** Department of Environmental Medicine, New York University School of Medicine, 650 First Avenue Room 539, New York, NY, 10016–3240, USA; Kaplan Comprehensive Cancer Center, New York University School of Medicine, New York, USA; Department of Obstetrics and Gynecology, New York University School of Medicine, New York, 550 First Avenue NB9E2, New York, NY, 10016, USA; Department of Pathology, Karmanos Cancer Institute Wayne State University, 110 E Warren Ave., Detroit, MI, USA; Department of Pathology, New York University School of Medicine, 550 First Avenue NB 4 West 35B, New York, NY, 10016, USA

**Keywords:** endometrial cancer, oestrogen, oestradiol, SHBG, nested case–control study, body mass index

## Abstract

We assessed the association of postmenopausal serum levels of oestrogens and sex hormone-binding globulin (SHBG) with endometrial cancer risk in a case–control study nested within the NYU Women's Health Study cohort. Among 7054 women postmenopausal at enrolment, 57 cases of endometrial cancer were diagnosed a median of 5.5 years after blood donation. Each case was compared to 4 controls matched on age, menopausal status at enrolment, and serum storage duration. Endometrial cancer risk increased with higher levels of oestradiol (odds ratio = 2.4 in highest vs lowest tertile, *P* for trend = 0.02), percent free oestradiol (OR = 3.5, *P*< 0.001), and oestrone (OR = 3.9, *P*< 0.001). Risk decreased with higher levels of percent SHBG-bound oestradiol (OR = 0.43, *P* = 0.03) and SHBG (OR = 0.39, *P* = 0.01). Trends remained in the same directions after adjusting for height and body mass index. A positive association of body mass index with risk was substantially reduced after adjusting for oestrone level. Our results indicate that risk of endometrial cancer increases with increasing postmenopausal oestrogen levels but do not provide strong support for a role of body mass index independent of its effect on oestrogen levels. © 2001 Cancer Research Campaign http://www.bjcancer.com


*British Journal of Cancer* (2001) **84**(7), 975–981
© 2001 Cancer Research Campaign
doi: 10.1054/ bjoc.2001.1704, available online at http://www.idealibrary.com on IDEAL®

http://www.bjcancer.com

### Postmenopausal endogenous oestrogens and risk of endometrial cancer: results of a prospective study

**A Zeleniuch-Jacquotte[1,2], A Akhmedkhanov[1,3], I Kato[4], KL Koenig[1,2], RE Shore[1,2], MY Kim[1,2], M Levitz[3], KR Mittal[5], U Raju[3], S Banerjee[3] and P Toniolo[1,2,3]**

[1]Department of Environmental Medicine, New York University School of Medicine, 650 First Avenue, Room 539, New York, NY 10016–3240, USA; [2]Kaplan Comprehensive Cancer Center, New York University School of Medicine, New York, USA; [3]Department of Obstetrics and Gynecology, New York University School of Medicine, New York, 550 First Avenue NB9E2, New York, NY 10016, USA; [4]Karmanos Cancer Institute, Department of Pathology Wayne State University, 110 E Warren Ave., Detroit MI, USA; [5]Department of Pathology, New York University School of Medicine, 550 First Avenue NB 4 West 35B, New York, NY 10016, USA

**Summary** We assessed the association of postmenopausal serum levels of oestrogens and sex hormone-binding globulin (SHBG) with endometrial cancer risk in a case–control study nested within the NYU Women's Health Study cohort. Among 7054 women postmenopausal at enrolment, 57 cases of endometrial cancer were diagnosed a median of 5.5 years after blood donation. Each case was compared to 4 controls matched on age, menopausal status at enrolment, and serum storage duration. Endometrial cancer risk increased with higher levels of oestradiol (odds ratio = 2.4 in highest vs lowest tertile, $P$ for trend = 0.02), percent free oestradiol (OR = 3.5, $P < 0.001$), and oestrone (OR = 3.9, $P < 0.001$). Risk decreased with higher levels of percent SHBG-bound oestradiol (OR = 0.43, $P = 0.03$) and SHBG (OR = 0.39, $P = 0.01$). Trends remained in the same directions after adjusting for height and body mass index. A positive association of body mass index with risk was substantially reduced after adjusting for oestrone level. Our results indicate that risk of endometrial cancer increases with increasing postmenopausal oestrogen levels but do not provide strong support for a role of body mass index independent of its effect on oestrogen levels. © 2001 Cancer Research Campaign http://www.bjcancer.com

**Keywords**: endometrial cancer; oestrogen; oestradiol; SHBG; nested case–control study; body mass index

The prevailing theory regarding the development of endometrial cancer is the 'unopposed oestrogen' theory, that proposes that endometrial cancer risk is increased by exposure to oestrogens unopposed by progesterone and that this increased risk is a result of increased mitotic activity of the endometrium (Siiteri, 1978; Henderson et al, 1982). The theory is based on epidemiologic observations, as well as observations on the hormonal regulation of endometrial cell mitoses during normal menstrual cycles. The endometrial cell division rate appears to be maximally stimulated by the low levels of oestradiol found in the early follicular phase and to be effectively zero in the presence of luteal phase progesterone (Ferenczy et al, 1979).

The unopposed oestrogen hypothesis successfully explains many of the epidemiologic features of the disease, such as the increased risks associated with oestrogen-secreting tumours, and with use of oestrogen replacement therapy. The hypothesis is also consistent with the reduction in risk observed with use of combined oral contraceptives, which are associated with minimal endometrial exposure to unopposed oestrogens and rare cell division. In addition, the unopposed oestrogen hypothesis provides a plausible mechanism for postmenopausal obesity, which, with oestrogen replacement therapy, is the most important risk factor for endometrial cancer. Production of oestrogens through aromatization of androgens in peripheral tissues, particularly in adipose tissue, is the main source of oestrogens in postmenopausal women.

Whereas the role of exogenous oestrogens in the development of endometrial cancer is well established, less epidemiologic evidence is available regarding the role of endogenous oestrogens, in particular at the physiologically low levels observed after menopause. An upper limit has been proposed by Key and Pike, (1988) beyond which oestradiol unopposed by progesterone does not induce any further increase in mitotic rate, and estimated this limit to be 50 pg $ml^{-1}$, the upper limit of oestradiol during the early follicular phase of the menstrual cycle. After menopause, levels of oestradiol are well below this level, in the range of 5 to 20 pg $ml^{-1}$, and progesterone levels are also low, 40 ng $dl^{-1}$ or less. According to the Key and Pike hypothesis, one would therefore expect postmenopausal serum levels of oestrogens to be associated with risk of endometrial cancer.

A number of case–control studies have examined the association of endogenous oestrogen levels with endometrial cancer risk (Aleem et al, 1976; Benjamin and Deutsch, 1976; Judd et al, 1976, 1980; Nisker et al, 1980; Davidson et al, 1981; von Holst et al, 1981; Bremond et al, 1982; Jasonni et al, 1984; Gimes et al, 1986; Petterson et al, 1986; Austin, 1991; Nyholm, 1993; Postischman, 1996). However, a limitation of these studies is that hormone measurements were made in blood samples drawn after diagnosis, and may have been influenced by the presence of the tumour. We report here on a case–control study of endometrial cancer nested within the New York University (NYU) Women's Health Study, a prospective cohort study of hormonal and environmental factors and cancer in women (Toniolo et al, 1991, 1995). This study is, to

*Received 27 September 2000*
*Revised 16 January 2001*
*Accepted 3 January 2001*

*Correspondence to:* A Zeleniuch-Jacquotte our knowledge, the first prospective study of endogenous oestrogens and risk of endometrial cancer. We also examined the association of body mass index with risk of endometrial cancer, and the effect of adjusting for oestrogen levels on this association.

### MATERIAL AND METHODS

### The NYU Women's Health Study

Between 1985 and 1991, the NYU Women's Health Study enrolled 14 275 healthy women aged 34–65 years at the Guttman Breast Diagnostic Institute, a breast cancer screening centre in New York City (Toniolo et al, 1991, 1995). Among the 7054 cohort members who were postmenopausal at the time of enrolment, 1641 had had a hysterectomy. The current report is limited to the remaining 5413 postmenopausal participants. Women were classified as postmenopausal at entry if they had had no menstrual cycles during the 6 months preceding enrolment, or if they had had a total bilateral oophorectomy. Women who had taken hormonal medications in the 6 months preceding their visit were not eligible. After written informed consent was obtained, demographic, medical, anthropometric, reproductive, and dietary data were collected through self-administered questionnaires. 30 millilitres of non-fasting peripheral venous blood were drawn prior to breast examination. After centrifugation, serum samples were divided into 1 millilitre aliquots and immediately stored at –80˚C for subsequent biochemical analyses. Up to 1991, women who returned for annual breast cancer screening were invited to contribute additional blood donations.

### Nested case–control study

Cases of endometrial cancer were identified through active follow-up of the cohort by mailed questionnaires every 2 years and telephone interviews for non-respondents, as well as record linkage with state cancer registries in New York, New Jersey, and Connecticut, and with the National Death Index. A capture–recapture analysis estimated that the ascertainment rate for breast cancer in our cohort is 95% (Kato et al, 1999). Medical and pathology reports were requested to confirm the diagnosis. Cases diagnosed within 6 months of blood donation were excluded in order to reduce the potential for observing differences in hormonal concentrations due to the tumour growth.

For each case subject, 4 controls were selected at random from the risk set of women who were alive and free of disease at the time of diagnosis of the case (index date), who had not had hysterectomy prior to the index date, and who matched the case on menopausal status at enrolment, date of birth (± 6 months), and number (1, > 1) of blood donations. For women who gave blood more than once samples from the second blood donation were used, but only if the lagtime between second blood donation and endometrial cancer diagnosis was at least 6 months. The policy of the NYU Women's Health Study is to use the second blood donation whenever possible to allow the first blood donation to be preserved for future studies of other diseases which may have been diagnosed between the first and second blood samplings. Controls were matched to cases on the date of first blood donation (± 3 months) if the first serum sample was used, or second blood donation (± 3 months) otherwise. Relaxation criteria (date of birth ± 12 months and date of blood donation ± 6 months) were used if the risk set for a case had less than 4 subjects. Both case and control subjects who had reported breast cancer prior to the index date were excluded because of the common use of tamoxifen in the treatment of breast cancer and the well known increase in risk of endometrial cancer associated with use of this therapeutic agent.

Cases and controls were contacted by telephone to collect detailed information on endometrial cancer risk factors. Data were collected on medical history, smoking, oral contraceptive and hormone replacement therapy use up to the index date. A calendar to record reproductive life events and two photo booklets (one for oral contraceptives and one for hormone replacement therapy) were mailed to each participant prior to the interview. The Nurses' Health Study photo booklet for oral contraceptives was used and a photo booklet for hormone replacement therapy was developed specifically for this study. Interviewers were unaware of the case or control status of the interviewees.

### Laboratory analyses

Percent oestradiol bound to SHBG was determined by a Concanavalin A-agarose binding assay and percent oestradiol free by an ultrafiltration method, as described previously (Toniolo et al, 1995). SHBG was quantified by a chemiluminescent immunometric assay using immunolite technology (Diagnostic Products Corp, Los Angeles, CA). The assays for total oestradiol and oestrone were performed by the Clinical Studies Center of Quest Diagnostics Inc (Nichols Institute, San Juan Capistrano, CA). Serum samples were submitted to organic extraction and celite chromatography and the appropriate fractions analysed by radioimmunoassay (Abraham et al, 1970; Mikhail and Chung, 1970). The quantity of serum was insufficient to determine oestrone levels for 8 women (1 case and 7 controls) and oestradiol levels for 4 women (all controls). These women were excluded from the analyses of the hormone for which they had missing data. However, because most of them were controls and because a matched set was composed of one case and 4 controls, no matched set was excluded from the oestradiol analysis and only one matched set was excluded from the oestrone analyses. In 11 samples, levels were below the detection limit of oestrone (10 pg $ml^{-1}$) and were arbitrarily set at 5 pg $ml^{-1}$. For oestradiol, the detection limit was 2 pg $ml^{-1}$ and for SHBG 0.2 nmol $ml^{-1}$; none of the measurements were below these levels.

Laboratory personnel were not aware of the case or control status of the samples, and samples from a case and her matched controls were analysed in the same batch. In order to assess laboratory precision, one aliquot from a common pool was included with every other matched set after labelling to preclude identification. The pool was generated using serum samples from a random sample of the healthy postmenopausal NYU Women's Health Study participants. Intra-batch coefficients of variation were 4.8% for total oestradiol, 9.5% for oestrone, 16.0% for percent free oestradiol, 4.2% for percent oestradiol bound to SHBG, and 6.5% for SHBG. The median storage time was 12.3 years (range, 8.3–13.8 years).

### Statistical methods

Hormone and SHBG measurements were log-transformed to reduce departures from the normal distribution with the exception of percent free oestradiol and percent SHBG-bound oestradiol. Case and control hormonal levels were compared using a mixed effects linear regression model to take into account the matched design (Liang and Zeger, 1986).

© 2001 Cancer Research Campaign

To compute odds ratios, serum measurements were categorized into tertiles, using the frequency distribution of the cases and the controls combined. The data were analysed using conditional logistic regression (Breslow and Day, 1980). Odds ratios were computed relative to the lowest tertile. Likelihood ratio tests were used to assess the significance of overall associations, linear trends and deviations from linearity. Reported trend test *P* values correspond to hormone and SHBG variables treated as ordered categorical variables. Analyses were also performed on the continous variables. All *P* values are two-sided.

Because of the known association between obesity and oestrogen levels, we conducted analyses adjusting for body mass index assessed at time of blood donation. In these analyses, height was also included in the models because it has been shown that, if obesity is a risk factor, including only body mass index in a model forces an inverse-quadratic relation of height to risk given weight (Michels et al, 1998). The effect of other potential confounders was explored by examining the association of these factors with disease and hormone levels. The variations in the hormone regression coefficients induced by the addition of the potential confounders in the logistic regression models were also examined. Potential confounders considered were nulliparity (collected at enrolment), history of diabetes, smoking history, oral contraceptive use and hormone replacement therapy use up to index date (collected by telephone interviews).

Logistic regression parameter estimates were corrected for measurement error in the hormone and BMI variables using the error-correction method of Armstrong et al (Armstrong et al, 1989) for matched case–control data. Within-subject variance estimates for percent oestradiol free, percent oestradiol bound to SHBG and BMI were based on results from our previous reliability studies (Sonnenschein et al, 1993; Toniolo et al, 1994). For log oestradiol and log oestrone, estimates of within-subject variance were provided by S Hankinson (personal communication, 2000). These data came from a reliability study conducted in 79 healthy postmenopausal Nurses' Health Study participants who had given 3 annual blood samples (Hankinson et al, 1995). Assays were conducted in the same laboratory as for our study, i.e. the Nichols Institute. Error terms associated with each exposure variable were assumed to be independent of each other, of the true value of the exposure, and of measurement errors from other exposure variables.

### RESULTS

By December 31, 1997, 63 participants postmenopausal at enrolment had been diagnosed with endometrial cancer. Pathology reports were obtained for 90% of the cases; tumour confirmation came from the tumour registries for the remaining 10%. 4 cases were excluded because they had been diagnosed with breast cancer prior to endometrial cancer and 2 cases because of the histology of their tumour (one leiomyosarcoma and one mesodermal mixed tumour of the uterus). 80% of the tumours were endometrioid carcinomas, 12% were papillary serous adenocarcinoma, and the remainder were mucinous or could not be classified further due to lack of specification on the pathology report. 6 of the controls originally selected were excluded (4 had been erroneously classified as postmenopausal at entry and 2 had died before the index date). One participant who was selected as a control became a case later on and was included in both matched sets as recommended when risk-set sampling is used (Lubin and Gail, 1984). As a result, 57 cases and 222 controls are included in the present report. Relaxation criteria were used to select 15 (7%) of the controls. Interviews were obtained for 235 participants, resulting in a response rate of 84% (88% for cases and 83% for controls). For 18 of the participants (12 cases, i.e. 24%, and 6 controls, i.e. 3%) the interview was completed by a surrogate. For 3 cases and 29 controls who had not completed the telephone interview, information on some risk factors for endometrial cancer (history of diabetes, smoking, oral contraceptive use, hormone replacement therapy use) was available from a previous follow-up questionnaire. As a result, data were available on 53 cases (93%) and 213 controls (96%) for most risk factors.

The median age at blood donation was 61.1 years and the median age at diagnosis of endometrial cancer was 65.7 years (Table 1). The median body mass index was significantly higher ($P < 0.01$) for case participants (25.5 kg/m$^2$) than for control participants (24.7 kg/m$^2$). Ever use of hormone replacement therapy and history of diabetes were reported more often by case than by control subjects whereas ever smoking was more common in controls than in cases. These differences were not statistically significant. One control and none of the cases reported total bilateral oophorectomy prior to entering the study.

Table 2 reports serum levels of oestrogens and SHBG. Total oestradiol, percent free oestradiol, and oestrone were significantly

**Table 1** Characteristics of postmenopausal endometrial cancer case and control subjects, NYU Women's Health Study, 1985–1997

| | Cases (*n* = 57) | Controls (*n* = 222) |
|---|---|---|
| Age (years) at blood donation, median (range) | 61.1 (45.2–66.0) | 61.1 (44.0–67.1) |
| Age (years) at diagnosis, median (range) | 65.7 (47.7–75.3) | |
| Height (cm), median (range) | 162.7 (147–183) | 160.1 (147–178) |
| Weight (kg)*, median (range) | 67.2 (46.8–113.5) | 63.6 (45.4–109.0) |
| Body mass index (kg/m$^2$)*, median (range) | 25.8 (17.7–44.2) | 24.7 (17.7–37.8) |
| Nulliparous[1], *n* (%) | 12 (24%) | 53 (26%) |
| Ever used oestrogen or oestrogen + progestagen replacement therapy[1], *n* (%) | 17 (32%) | 53 (25%) |
| Ever used oestrogen alone replacement therapy[1], *n* (%) | 10 (20%) | 30 (16%) |
| Ever used oral contraceptives[1], *n* (%) | 8 (15%) | 37 (17%) |
| History of diabetes[1], *n* (%) | 6 (11%) | 13 (6%) |
| Ever smoker[1], *n* (%) | 21 (42%) | 95 (52%) |

*$P$ < 0.01. [1]Data missing for some subjects.

**Table 2** Median, 10th and 90th percentiles of serum levels of oestrogens and SHBG in postmenopausal endometrial cancer case and controls subjects, NYU Women's Health Study, 1985–1997

| | Cases (*n* = 57) | Controls (*n* = 222) |
|---|---|---|
| Total oestradiol**, pg ml$^{-1}$ | 8 (4–22) | 7 (4–12) |
| Free oestradiol**, % | 1.31 (0.94–1.50) | 1.17 (0.95–1.44) |
| SHBG-bound oestradiol**, % | 36.7 (19.6–51.9) | 39.5 (27.2–50.6) |
| Oestrone**, pg ml$^{-1}$ | 27 (14–59) | 22 (12–38) |
| SHBG*, nMol L$^{-1}$ | 45.3 (21.4–95.2) | 55.3 (31.2–92.9) |

*$P$ = 0.05; ** $P$ < 0.01.

higher in case than in control subjects. In addition, SHBG and percent SHBG-bound oestradiol were significantly lower in cases than in controls. Odds ratios for endometrial cancer were consistent with these results (Table 3). An increase in risk was observed with higher levels of oestradiol (OR = 2.4 in the highest versus the lowest tertile, *P* for trend = 0.02), percent free oestradiol (OR = 3.5 in highest tertile, *P* for trend < 0.001), and oestrone (OR = 3.9 in highest tertile, *P* for trend < 0.001). A decrease in risk was observed with higher levels of percent oestradiol bound to SHBG (OR = 0.43 in highest tertile, *P* for trend = 0.03) and SHBG (OR = 0.39 in highest tertile, *P* for trend = 0.01). All these associations were reduced after adjustment for body mass index and height. The associations with oestradiol and percent oestradiol bound to SHBG were no longer significant and the association with SHBG was only marginally significant (Table 3). None of the tests for deviation from linearity was significant and results using continuous variables were similar to results using tertiles. The regression coefficients adjusted for height and body mass index were 0.63 (standard error = 0.30, *P* value = 0.04) for $\log_{10}$ (oestradiol), 2.75 (standard error = 0.95, *P* value = 0.003) for percent free oestradiol, –0.033 (standard error = 0.018, *P* value = 0.06) for percent SHBG-bound oestradiol, 0.69 (standard error = 0.34, *P* value = 0.04) for $\log_{10}$ (oestrone), and –0.56 (standard error = 0.34, *P* value = 0.09) for SHBG, respectively.

The effect of other potential confounders was explored. There was a slightly lower proportion of ever smokers in cases (42%) than in controls (52%), and a history of smoking was associated with lower levels of oestradiol (6.4 pg ml$^{-1}$ for ever smokers versus 7.5 pg ml$^{-1}$ for never smokers, *P* = 0.01) and higher levels of SHBG (56.5 nMol l$^{-1}$ for ever smokers versus 49.0 nMol l$^{-1}$ for never smokers, *P* = 0.01) in control subjects. However, adjusting for smoking history had very little impact on the logistic regression coefficients associated with hormones and SHBG. For instance the regression coefficient for oestradiol went from 0.58 (standard error 0.335) in the model adjusting for height and body mass index to 0.56 (standard error 0.336) in the model in which smoking was added. Other potential risk factors (nulliparity, use of hormone replacement therapy, use of oral contraceptives, and history of diabetes) showed no association with hormone and SHBG levels, and had no material impact on the logistic regression coefficients for hormones and SHBG. Because of the lack of evidence of confounding effects, and because adjusting for these factors resulted in a smaller study size due to missing data on some of the subjects, odds ratios adjusting only for height and body mass index are presented.

**Table 3** Odds ratios and 95% confidence intervals for endometrial cancer associated with levels of hormones and sex hormone-binding globulin (SHBG) among postmenopausal women: NYU Women's Health Study, 1985–1997

| | No. cases (%) | No. controls (%) | Unadjusted OR[1] (95% CI) | Adjusted OR[2] (95% CI) |
|---|---|---|---|---|
| Oestradiol, pg ml$^{-1}$ | | | | |
| < 6 | 14 (24.6%) | 77 (35.3%) | 1.0 | 1.0 |
| 6–8 | 16 (28.1%) | 80 (36.7%) | 1.1 (0.52–2.4) | 1.0 (0.47–2.3) |
| > 8 | 27 (47.4%) | 61 (28.0%) | 2.4 (1.1–4.9) | 1.8 (0.75–4.2) |
| *P* for trend | | | 0.02 | 0.19 |
| Free oestradiol, % | | | | |
| < 1.10 | 13 (22.8%) | 78 (35.1%) | 1.0 | 1.0 |
| 1.10–1.28 | 13 (22.8%) | 84 (37.8%) | 1.1(0.44–2.4) | 0.91 (0.38–2.2) |
| > 1.28 | 31 (54.4%) | 60 (27.0%) | 3.5 (1.6–7.5) | 2.8 (1.3–6.4) |
| *P* for trend | | | < 0.001 | 0.004 |
| SHBG-bound oestradiol, % | | | | |
| < 35.26 | 25 (43.9%) | 68 (30.6%) | 1.0 | 1.0 |
| 35.26–43.30 | 18 (31.6%) | 76 (34.2%) | 0.59 (0.28–1.2) | 0.66 (0.31–1.4) |
| > 43.30 | 14 (24.6%) | 78 (35.1%) | 0.43 (0.20–0.95) | 0.60 (0.26–1.4) |
| *P* for trend | | | 0.03 | 0.22 |
| Oestrone, pg ml$^{-1}$ | | | | |
| < 20 | 10 (17.9%) | 83 (38.6%) | 1.0 | 1.0 |
| 20–28 | 19 (33.9%) | 76 (35.3%) | 2.1 (0.9–4.8) | 2.0 (0.9–4.7) |
| > 28 | 27 (48.2%) | 56 (26.0%) | 3.9 (1.8–8.7) | 3.2 (1.3–7.8) |
| | | | <0.001 | 0.008 |
| SHBG, nMol l$^{-1}$ | | | | |
| < 44.3 | 26 (45.6%) | 67 (30.3%) | 1.0 | 1.0 |
| 44.3–64.4 | 18 (31.6%) | 74 (33.5%) | 0.60 (0.30–1.2) | 0.67 (0.33–1.4) |
| > 64.4 | 13 (22.8%) | 80 (36.2%) | 0.39 (0.18–0.84) | 0.49 (0.22–1.1) |
| *P* for trend | | | 0.01 | 0.08 |

[1]Except for matching factors. [2]For In (height) and In (body mass index).

© 2001 Cancer Research Campaign

### DISCUSSION

The median lagtime between blood donation and diagnosis in our study was 5.5 years (range 0.5–11.4 years). 47 (82%) of the tumours were diagnosed at least 2 years after blood donation. To our knowledge, this is the first prospective study to report on the association of oestrogen levels with endometrial cancer risk. Higher levels of oestrone, total oestradiol, and percent free oestradiol were associated with an increased risk, whereas higher levels of SHBG and SHBG-bound oestradiol were associated with a decreased risk. Although some of the associations were no longer statistically significant after adjusting for height and body mass index, trends remained in the same directions and the limited sample size may have contributed to the loss of significance.

All women were postmenopausal with a median age at blood donation of 61 years (range 44–67 years), and the levels of oestrogens were in the low range expected in this age group (median oestradiol in the control group: 7 pg $ml^{-1}$, 10–90th percentiles, 4–12 pg $ml^{-1}$). Our results are consistent with the prediction of Key and Pike (Key and Pike, 1988) that an increase in risk would be observed with higher oestrogen levels, within the physiologically low menopausal range of levels.

Early case–control studies, involving limited numbers of cases, showed inconsistent results (Aleem et al, 1976; Benjamin and Deutsch, 1976; Judd et al, 1976, 1980; Nisker et al, 1980; Davidson et al, 1981; von Holst et al, 1981; Bremond et al, 1982; Jasonni et al, 1984; Gimes et al, 1986; Pettersson et al, 1986). 3 more recent and larger case–control studies have reported positive associations with postmenopausal levels of oestrogens. In a study of 168 cases and 334 controls (Austin et al, 1991), cases had statistically significantly higher serum oestrone and oestradiol levels than controls, while another study (Nyholm et al, 1993) reported higher levels of oestradiol, free oestradiol and oestrone in 50 cases than in 54 matched controls. The largest case–control study to date (Potischman et al, 1996), conducted in 5 US geographic regions, with 208 postmenopausal matched case–control pairs observed an increased risk of endometrial cancer with higher levels of total oestradiol (geometric mean in cases = 10.8 pg $ml^{-1}$ versus 7.4 pg $ml^{-1}$ in controls), oestrone (geometric mean in cases = 43.7 pg $ml^{-1}$ versus 33.1 pg $ml^{-1}$ in cases), free oestradiol, albumin-bound oestradiol, and oestrone sulphate. A trend of reduced risk was observed with increasing levels of SHBG (geometric mean in cases = 30.6 pg $ml^{-1}$ versus 41.5 pg $ml^{-1}$ in controls).

The study of Potischman et al (1996) also reported a strong association with obesity, with an odds ratio of 3.8 for a body mass index of 30.0 kg/$m^2$ or more compared with one lower than 23.0 kg/$m^2$. The odds ratio was slightly reduced (to 3.1) but remained significant after adjusting for oestrone levels and correcting for measurement error (Potischman et al, 1999). This result was unexpected because body mass index is generally thought to play a role in the development of endometrial cancer through increased production of oestrogens via peripheral aromatization of androgens. Its association with endometrial cancer risk would therefore be expected to be substantially reduced when adjusting for oestrogen levels, unless it acts through some other mechanisms. In Table 4, we report the logistic regression coefficients for endometrial cancer associated with body mass index in an analysis similar to that mentioned above (Potischman et al, 1999). Prior to adjustment for hormone level, risk showed a significant positive association with body mass index (conditional logistic regression coefficient = 0.087, $P$ = 0.007), this was substantially reduced and no longer significant after adjusting for oestrone level (regression coefficient = 0.051, $P$ = 0.16). These results are similar to those of Potischman et al with respect to the direction, but not the strength, of the association: we found the association with body mass index to be weaker (regression coefficient 0.051 versus 0.081), and with oestrone stronger (regression coefficient 0.68 versus 0.44). In both studies, correcting for measurement error resulted in an increase of the oestrone coefficient and a small attenuation of the body mass index coefficient. Although our results do not refute a role of body mass index in endometrial cancer independently of its effect on oestrogen levels, they do not provide the same support for this hypothesis as the other study (Potischman et al, 1999).

A limitation of our study is that data on use of oral contraceptives and hormone replacement therapy, 2 factors strongly associated with endometrial cancer risk and therefore potential confounders, were collected after diagnosis of the cases, raising the concern of recall bias. In addition, for deceased participants, telephone interviews were completed by surrogates who may not have had accurate information about these exposures. Because more case than control interviews were completed by surrogates, differential misclassification may have resulted. For a factor to confound an association, though, it needs to be associated with the exposures of interest, here postmenopausal oestrogen and SHBG levels, in addition to being associated with disease. Women who use hormone replacement therapy tend to be leaner, suggesting that they may have lower oestrogen levels than women who do not use them (Derby et al, 1993). However, in our dataset there was no evidence of a difference in age-adjusted levels of sex hormones or SHBG between control subjects who had and had not used hormone replacement therapy. For instance, for oestradiol geometric means were 7.2 pg $ml^{-1}$ in users and 6.7 in nonusers ($P$ = 0.17); for oestrone the geometric means were 20.4 pg $ml^{-1}$ and 21.5 pg $ml^{-1}$ in users and nonusers respectively ($P$ = 0.50). Regarding oral contraceptive use, there are no data to suggest an association with subsequent postmenopausal endogenous

**Table 4** Logistic regression coefficients for endometrial cancer associated with In (oestrone) and body mass index in the NYU Women's Health Study and the Potischman et al study (Potischman et al, 1999)

| | NYU Women's Health Study | | | Potischman et al study | |
|---|---|---|---|---|---|
| | Unadjusted | Adjusted[1] | Adjusted and corrected[2] | Adjusted[1] | Adjusted and corrected[2] |
| In (oestrone) | 0.88 | 0.68 | 0.81 | 0.44 | 0.62 |
| Body mass index | 0.087 | 0.051 | 0.048 | 0.081 | 0.075 |

[1]Adjusted for the other variable in the table. [2]Adjusted for the other variable in the table and corrected for measurement error.

oestrogen levels. These results suggest that confounding by these factors was unlikely in our study. It should be noted that the prevalence of both oral contraceptive use and hormone replacement therapy were low in our cohort because women who had used either one of these in the 6 months preceding enrolment were not eligible. The major strength of our study is that the serum samples used for hormone measurements were collected prior to disease and that no selection bias was operating because these measurements were available on all cases and controls.

There is increasing evidence that oestrogen may be associated only with some types of endometrial cancer, i.e. endometrioid adenocarcinomas, also called type I carcinomas. Type II carcinomas that include serous carcinomas and some other aggressive types are observed in older women, develop from atrophic rather than hyperplastic epithelium and do not appear to be related to oestrogen exposure (Sherman, 2000). Because of the limited sample size of our study and the lack of specification of some of the pathology reports, we were not able to examine separately the different histopathologic types. Future epidemiologic studies should do so, although the lack of standardization of pathological reporting and the relatively small proportion of type II carcinomas (approximately 20%), may hamper such efforts.

In conclusion, postmenopausal levels of oestrone, total oestradiol, and percent-free oestradiol, measured 6 months to 11 years prior to diagnosis, were positively associated with risk of endometrial cancer in our study. SHBG and percent SHBG-bound oestradiol were negatively associated with risk. Body mass index did not seem to be associated with risk of endometrial cancer after adjusting for oestrone levels.

### ACKNOWLEDGEMENTS

Supported by grants CA66189, CA34588, and CA16087 from the National Cancer Institute, National Institutes of Health. We thank Dr Louise A Brinton for providing a copy of the questionnaire used in a multi-centre case–control study of endometrial cancer that we used as a model in the development of our own questionnaire, Dr Susan Hankinson for the unpublished data used in our analysis correcting for error in measurement, and Dr Walter C Willett for providing the Nurses' Health Study oral contraceptives booklets. We also thank Samantha Garbers for her help in developing the hormone replacement therapy booklet. We thank Nicole Heitler and Joyce Hansen who conducted all the telephone interviews, and Jo-Ann Cutrone, Daniela Masciangelo, and Lynne Quinones for technical assistance.

### REFERENCES

Abraham GE, Tulchinsky D and Korenman SG (1970) Chromatographic purification of estradiol 17B for use in radio-ligand assay. *Biochem Med* **3**: 365–368

Aleem FA, Moukhtar MA, Hung HC and Romney SL (1976) Plasma extrogen in patients with endometrial hyperplasia and carcinoma. *Cancer* **38**: 2101–2104

Armstrong B, Whittemore A and Howe G (1989) Analysis of case–control data with covariate measurement error: application to diet and colon cancer. *Stat Med* **8**: 1151–1163

Austin H, Austin JM, Partridge EE, Hatch KD and Shingleton HM (1991) Endometrial cancer, obesity and body fat distribution. *Cancer Res* **51**: 568–572

Benjamin F and Deutsch S (1976) Plasma levels of fractionated estrogens and pituitary hormones in endometrial carcinoma. *Am J Obstet Gynecol* **126**: 638–647

Bremond AG, Claustrat B, Rudigoz RC, Seffert P and Corniau J (1982) Estradiol, androstenedione, testosterone and dehydroepiandrosterone sulfate in the ovarian and peripheral blood of postmenopausal patients with and without endometrial cancer. *Gynecol Oncol* **14**: 119–124

Breslow NE and Day NE (1980) *Statistical Methods in Cancer Research volume 1*. Vol. 1. IARC Scientific Publications No. 32: Lyon

Davidson BJ, Gambone JC, Lagasse LD, Castaldo TW, Hammond GL, Sitteri PK and Judd HL (1981) Free estradiol in postmenopausal women with and without endometrial cancer. *J Clin Endocrinol Metab*, **52**: 404–408

Derby CA, Lamont Hume A, McFarland Barbour M, McPhillips JB, Lasater TM and Carleton RA (1993) Correlates of postmenopausal estrogen use and trends through the 1980s in two southeastern New England communities. *Am J Epidemiol* **137**: 1125–1135

Ferenczy A, Bertrand G and Gelfand MM (1979) Proliferation kinetics of human endometrium during the normal menstrual cycle. *Am J Obstet Gynecol* **133**: 859–867

Gimes G, Szarvas Z and Siklosi G (1986) Endocrine factors in the etiology of endometrial carcinoma. *Neoplasma* **33**: 393–397

Hankinson SE, Manson JE, Spiegelman D, Willett WC, Longcope C and Speizer F (1995) Reproducibility of plasma hormone levels in postmenopauseal women over a 2–3 year period. *Cancer Epidemiol Biomarkers Prev*, **4**: 649–654

Henderson BE, Ross RK, Pike MC and Casagande JT (1982) Endogenous hormones as a major factor in human cancer. *Cancer Res* **42**: 3232–3239

Jasonni VM, Bulletti C, Franceschetti F, Bonavia M, Bolelli G, Ciotti P and Flamigni C (1984) Estrone sulphate plasma levels in postmenopausal women with and without endometrial cancer. *Cancer* **53**: 2698–2700

Judd HL, Lucas WE and Yen SSC (1976) Serum 17β-estradiol and estrone levels in postmenopausal women with and without endometrial cancer. *J Clin Endocrinol Metab* **43**: 272–278

Judd HL, Davidson BJ, Frumar AM, Shamonki IM, Lagasse LD and Ballon SC (1980) Serum androgens and estrogens in postmenopausal women with and without endometrial cancer. *Am J Obstet Gynecol* **136**: 859–871

Kato I, Toniolo P, Koenig K, Kahn A, Schymura M and Zeleniuch-Jacquotte A (1999) Comparison of active and cancer registry-based follow-up for breast cancer in a prospective cohort study. *Am J Epidemiol* **149**: 372–378

Key TJA and Pike MC (1988) The dose-effect relationship between unopposed estrogens and endometrial mitotic rate: Its central role in explaining and predicting endometrial cancer risk. *Br J Cancer* **57**: 205–212

Liang IK and Zeger SL (1986) Longitudinal data analysis using generalized linear models. *Biometrika* **73**: 13–22

Lubin JH and Gail MH (1984) Biased selection of controls for case-control analyses of cohort studies. *Biometrics* **40**: 63–75

Michels KB, Greenland S and Rosner BA (1998) Does body mass index adequately capture the relation of body composition and body size to health outcomes? *Am J Epidemiol* **147**: 167–172

Mikhail G and Chung HW (1970) Radioimmunoassay of plasma estrogens. Use of polymerized antibodies. In *Immunologic methods in steroid determination*, Peron, F. & Caldwell, B. (eds) pp. 113. Appleton-Century-Crofts: New York.

Nisker JA, Hammond GL, Davidson BJ, Frumar AM, Takaki NK, Judd HL and Siiteri PK (1980) Serum SHBG capacity and the percentage of free estradiol in postmenopausal women with and without endometrial carcinoma. *Am J Obstet* **138**: 637–642

Nyholm HCJ, Nielsen AL, Lyndrup J, Dreisler A, Hagen C and Haug E (1993) Plasma oestrogens in postmenopausal women with endometrial cancer. *Br J Obstet Gynaecol* **100**: 1115–1119

Pettersson B, Bergstrom R and Johansson EDB (1986) Serum estrogens and androgens in women with endometrial carcinoma. *Gynecol Oncol* **25**: 223–233

Pike MC, Peters RK, Cozen W, Probst-Hensch NM, Felix JC, Wan PC and Mack TM (1997) Estrogen-progestin replacement therapy and endometrial cancer. *J Natl Cancer Inst* **89**: 1110–1116

Potischman N, Hoover RN, Brinton LA, Siiteri PK, Dorgan JF, Swanson CA, Berman ML, Mortel R, Twiggs LB, Barrett RJ, Wilbanks GD, Persky V and Lurain JR (1996) Case-control study of endogenous steroid hormones and endometrial cancer. *J Natl Cancer Inst* **88**: 1127–1135

Potischman N, Gail MH, Troisi R, Wacholder S and Hoover RN (1999) Measurement error does not explain the persistence of a body mass index association with endometrial cancer after adjustment for endogenous hormones. *Epidemiol* **10**: 76–79

Sherman ME (2000) Theories of endometrial carcinogenesis: a multidisciplinary approach. *Mod Pathol* **13**: 295–308

Siiteri PK (1978) Steroid hormones and endometrial cancer. *Cancer Res* **38**: 4360–4366

Sonnenschein EG, Kim MY, Pasternack BS and Toniolo P (1993) Sources of variability in waist and hip measurements in middle- aged women. *Am J Epidemiol* **138**: 301–309

 *© 2001 Cancer Research Campaign*

Toniolo PG, Pasternack BS, Shore RE, Sonnenschein EG, Koenig KL, Rosenberg C, Strax P and Strax S (1991) Endogenous hormones and breast cancer: a prospective cohort study. *Breast Cancer Res Treat* **18**: S23–S26

Toniolo P, Koenig KL, Pasternack BS, Banerjee S, Rosenberg C, Shore RE, Strax P and Levitz M (1994) Reliability of measurements of total, protein-bound, and unbound estradiol in serum. *Cancer Epidemiol Biomarkers Prev* **3**: 47–50

Toniolo P, Levitz M, Zeleniuch-Jacquotte A, Banerjee S, Koenig KL, Shore RE, Strax P and Pasternack BS (1995) A prospective study of endogenous estrogens and breast cancer in postmenopausal women. *J Natl Cancer Inst* **87**: 190–197

von Holst T, Klinga K and Runnebaum B (1981) Hormone levels in healthy postmenopausal women and women with postmenopausal bleeding with and without endometrial carcinoma. *Maturitas*, **3**: 315–320

Weiderpass E, Adami H, Baron J, Magnusson C, Bergstrom R, Lindgren A, Correia N and Persson I (1999) Risk of endometrial cancer following estrogen replacement with and without progestins. *J Natl Cancer Inst* **91**: 1131–1137

© 2001 Cancer Research Campaign
